# It takes a community to help solve a cold case

**DOI:** 10.1016/j.fsisyn.2024.100482

**Published:** 2024-06-24

**Authors:** David A. Keatley

**Affiliations:** School of Law Murdoch University, Perth, Western Australia, Australia

They say it takes a village to raise a child; I believe it takes a community to help solve a cold case. Gathered in the following Special Issue is a representation of what the applied academic community can do, when we work together. Current major crimes pervade the daily news cycles are pervaded with current major crimes, which rightfully deserve our focus and attention to solve. But what of the older, colder cases? These cases, while sometimes decades old, still hold as much importance for the families affected and the police investigators still tirelessly working on them. The hope is that this special issue offers several avenues of support. Cold cases, a term that should arguably be confined to the history books, are ripe for reinvestigation. To call them cold is to underestimate the potential for reigniting the fire of investigative potential through new collaborations and methods. Several such methods are outlined here by academics from around the world who have contributed to the current special issue. Each paper shows the power of synergy, of a community, representing wider networks of support, expertise, and knowledge.

Understanding ‘what works’ in cold case investigations is central if we are to help investigators in the field. Toolin and colleagues focus primarily on psychological evidence-based research and present a systematic review of research regarding preventative methods and investigative techniques. The review highlights some of the promising leads in the field but casts a fair and scientific lens through which to understand, interpret, and progress the area moving forward. Their finding that in its present form psychological research is insufficient to inform evidence-based guidance should be seen as a caution to applied practitioners and a call-to-action for researchers. The onus is on us to continue developing our methods to improve their application.

A sign of this improved psychological application can be seen in the paper by Brooks and Hira, which outlines the role of operational psychology in New Zealand. Brooks and Hira outline key areas that operational psychology can be applied in cold cases, including, personality and offender profiling, crime analysis, victimology, interviewing, and equivocal death analysis, among others. Their paper provides a cautious yet optimistic outlook for the role and future of operational and forensic psychology in Behavioural Science Units focusing on cold cases.

Sadly, many cold cases are cold owing to the lack of a body. No-body homicides are some of the most challenging to investigate and resolve. The role of forensic science, with its myriad achievements, is rendered relatively obsolete if a body is not found. To this extent, two papers in this Special Issue outline current, cutting-edge approaches to finding the missing. First, Berezowski and colleagues provide a multidisciplinary approach to locating clandestine graves. Through the combined use of geographic profiling, LiDAR, and near surface geophysics, Berezowski and colleagues offer a promising account of what can be done to assist investigators. This multidisciplinary approach speaks to the heart of the power of multidisciplinary, multimethod synergy.

Investigators, however, may not always have the funds or time to use technology to scan large geographical areas. Narrowing the spotlight of a searchlight is sometimes required. In the second paper focusing on no-body homicides, Keatley provides a potential new lens to re-view cold cases. Taking the approach of ‘Winthropping’ – a method used to locate clandestine locations – Keatley suggests that focusing on forensic linguistics may offer insight through people's statements. Keatley shows several cases in which individuals potentially leaked information that related to where a body was later found. Though speculative and tentative in its nature, Keatley suggests that perhaps to find ‘where’ a body is hidden, we should study what may be hidden in people's words to see if they leak information in there.

Cold cases typically incur vast quantities of information, statements, tip-offs, conjectures, and hypotheses. Often it is not the lack of information that impedes a cold case review, but rather managing the synthesis and weighting of multiple hypothetical pathways. To assist with this, Keatley, Arntfield, and Clarke bring together the cutting-edge of temporal dynamics research for use in cold case reviews. Crime Script Sequencing – the combination of Crime Script Analysis and Behaviour Sequence Analysis – is outlined and applied to a real-world unsolved cold case. The authors show how while not a ‘solving’ tool, per se, it may provide some clarity to investigative pathways.

Finally, Guareschi and Magni provide an important overview of a real cold case. Guareschi and Magni shine a light on the real-world experience of working a cold case and the importance of attaining synergy between investigators, communities, and academics. Their conclusions provide a timely reminder of the need for synergy and community consideration. Therefore, to end where we began: it takes a whole community – including practitioners, academics, and the public – to solve a cold case, and this Special Issue provides several pathways forward.

